# Human papillomavirus infection and risk of lung cancer in never-smokers and women: an ‘adaptive’ meta-analysis

**DOI:** 10.4178/epih/e2015052

**Published:** 2015-11-17

**Authors:** Jong-Myon Bae, Eun Hee Kim

**Affiliations:** Department of Preventive Medicine, Jeju National University School of Medicine, Jeju, Korea

**Keywords:** Lung neoplasms, Risk factor, Human papillomavirus, Meta-analysis

## Abstract

**OBJECTIVES:**

The incidence of lung cancer in Koreans is increasing in women and in both men and women with a never-smoking history. Human papillomavirus (HPV) infection has been suggested as a modifiable risk factor of lung cancer in never-smokers and women (LCNSW). This systematic review (SR) aimed to evaluate an association between HPV infection and lung cancer risk in LCNSW.

**METHODS:**

Based on a prior SR and some expert reviews, we identified refereed, cited, or related articles using the PubMed and Scopus databases. All case-control studies that reported the odds ratio of HPV infection in LCNSW were selected. An estimate of the summary odds ratio (SOR) with 95% confidence intervals (CI) was calculated.

**RESULTS:**

A total of four case-control studies were included. The fixed-effect model was applied because of homogeneity (I-squared=0.0%). The SORs in women and in never-smokers were 5.32 (95% CI, 1.75 to 16.17) and 4.78 (2.25 to 10.15) respectively.

**CONCLUSIONS:**

These results showed a significant effect of HPV infection in LCNSW. It is evident that developing a preventive plan against LCNSW may be necessary.

## INTRODUCTION

Lung cancer ranks the first in cancer mortality in Korea and is the primary cancer with the heaviest disease burden [1]. According to the 2002-2012 statistics on lung cancer provided by Statistics Korea, the incidence rate in women increased and the rate of adenocarcinoma also increased during this time period [2]. These facts were corroborated by a study on lung cancer patients treated at a local cancer center [3], and particularly, the study authors reported that the majority of women with lung cancer were never-smokers (73.0%).

The increasing incidences of lung cancer among women never-smokers is a global trend [4,5], and it has been suggested that lung cancer in never-smokers should be considered separately, a disease different from lung cancer in smokers [6-8]. Among the hypotheses about the cause of lung cancer in women never-smokers, the one that has been given priority is second-hand smoke exposure [6,7,9-11]. However, a genome study has reported that the possibility of second-hand smoke involvement in lung cancer is low in Asian never-smokers [12]. In addition, second-hand smoke exposure also imposes a limit on cancer prevention efforts because control of second-hand smoke exposure cannot be achieved just by individuals making efforts, but requires efforts from society. Instead, a modifiable risk factor, human papillomavirus (HPV) infection, may be involved [6,7,9]. It is a risk factor for many cancers, such as cervical cancer [13], prostate cancer [14], and breast cancer [15], and currently, preventive vaccines are commercially available [16].

HPV deoxynucleic acid (DNA) is detected in approximately 20% of lung cancer tissues [17-20], and the detection rate is higher in lung cancer tissues of Asians [21-24]. The detection rate of HPV subtype 33 has been reported to be 31.3% among Koreans with lung cancer [25]. According to a systematic review reported in 2014 [26], the odds ratio (OR) of HPV infection was 5.67 (95% confidence interval [CI], 3.09 to 10.40), and among Asians it was 6.23 (95% CI, 2.78 to 13.97), showing a higher OR than for other races. However, this study did not present results from subgroup analysis on the OR of women and never-smokers. Thus, we have performed a systematic review that investigated the relationship between HPV infection and lung cancer in women and never-smokers.

## MATERIALS AND METHODS

### Search for and selection of relevant studies

In order to maximize the utility of previously conducted systematic reviews in the literature search, we used a manual search method rather than an automatic method [14,27,28]. Thus, we examined the references of five systematic review paper [17-20,26], and obtained, for each, the lists of “cited articles” and “similar (related) articles” provided by PubMed (www.ncbi.nlm.nih.gov/pubmed) and the Scopus (www.elsevier.com/solutions/scopus) database.

The finally selection criterion for the study objective was a case-control study in which HPV DNA was tested on never-smokers and women. Accordingly, for each study in the lists described above, the following exclusion criteria were applied to the abstract or the main body of the article: (1) a study dealing with different hypotheses, (2) an expert’s review or a systematic review study, and (3) a case report study. Of the remaining case-control studies after the first three exclusion criteria were applied, the final set of studies were selected using the following two exclusion criteria: (4) a study in which HPV DNA testing was not done on the pathological tissue, and (5) a study from which information on women or never-smokers cannot be obtained, even if it was a case-control study.

### Statistical analysis

Two researchers applied the exclusion criteria and obtained HPV-related information from each study. ORs and 95% CIs were computed for the confirmed patient groups and positive HPV values. Heterogeneity was evaluated using the I-squared values (%), and when homogeneity was confirmed, a meta-analysis was performed to compute summary odds ratios (SORs) and 95% CIs using a fixed-effect model. Statistical significance was determined at the level of 5%, and the statistical program Stata version 14.0 (Stata Corp., College Station, TX, USA) was used to conduct the analyses.

## RESULTS

**Figure 1.**
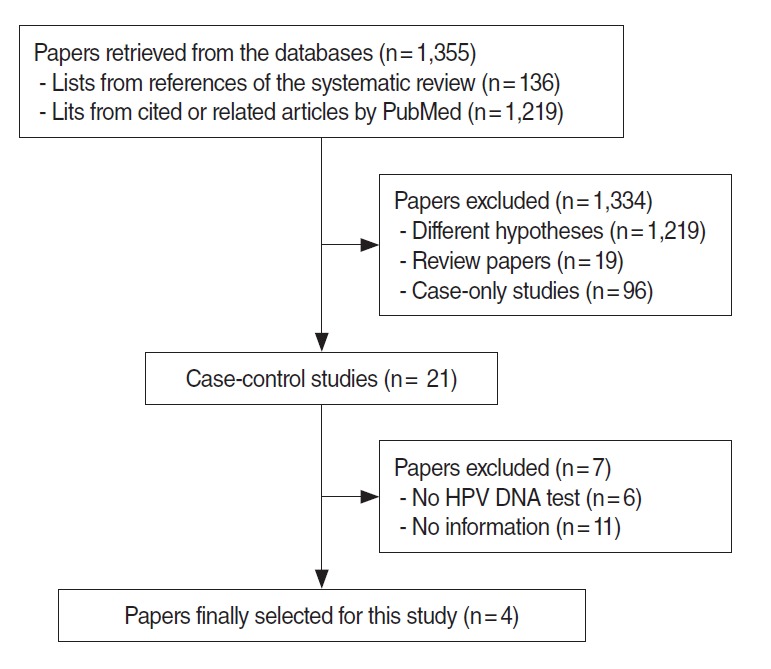
Flow chart of article selection. HPV, human papillomavirus; DNA, deoxynucleic acid.

Figure 1 shows the final study selection processes for the meta-analysis. We initially obtained 136 references from five systemic review papers and 1,219 cited and related studies through PubMed and Scopus. After the selection criteria were applied on a total of 1,355 studies, the number of studies excluded and the exclusion reasons were as follows: (1) 1,219 studies because they dealt with different hypotheses, (2) 19 studies because they were an expert’s review or a systematic review, (3) 96 case only studies, (4) six case-control studies that did not test for HPV DNA on the pathological tissue, and (5) 11 case-control studies in which information on women or never-smokers was not found. In summary, a total of 1,351 studies were excluded, and four studies were included [29-32].

**Figure 2.**
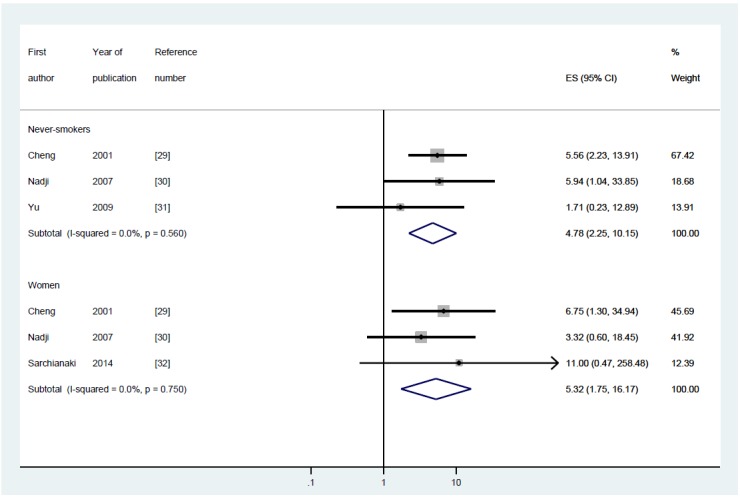
The forest plot of summary effect size (ES) with 95% confidence intervals (CI) using a fixed effect model by never-smokers and women.

Table 1 summarizes the four case-control studies, showing the nationality of the participants, test specimens, and distribution of participant groups of women and/or never-smokers, and the ORs and the 95% CIs computed depending on the presence or absence of positive HPV DNA. Information on a woman-only group and on a never-smoker group was presented in three studies each, and the I-squared value was 0% for both groups, suggesting homogeneity (Figure 2). Table 2 shows meta-analytic results from a fixed effect model on the HPV DNA subtypes 16/18. The SOR was 5.32 (95% CI, 1.75 to 16.17) in the women-only group and 4.78 (95% CI, 2.25 to 10.15) in the never-smoker group, showing a statistically significance.

## DISCUSSION

The SOR for lung cancer associated with HPV infection was 5.32 for the women and 4.78 for the never-smokers. These are at a level similar to SOR 5.67, which is an odds ratio computed for men and women together [26]. Considering that the risk of lung cancer of subtype 18 infection was reported to be 11.66-fold (95% CI, 2.94 to 46.27) in women never-smokers (Table 2) [29], the actual risk level for women never-smokers with HPV infection is expected to be higher. The findings provide evidence for the argument that vaccines against HPV infection can prevent the occurrence of lung cancer in women as well as protect against other cancers. In addition, from the finding that the SOR was similar when men and women were not separated and when participants were limited to women, HPV infection can be inferred to have a greater impact on the occurrence of lung cancer in women never-smokers than in men smokers [33,34].

The four studies selected in this systematic review [29-32] were all case-control studies. This is because of the difficulty in conducting a cohort study involving HPV DNA. Of the four included studies, three studies [29,30,32] were also included in Zhai et al.’s analysis [26]. The fourth study, Yu et al. [31], was selected instead of the one by the same authors presented in 2013 [35], because the former showed analytic results on women. The reason why additional studies were not included in the current systematic review is because the study closed the literature search in September 2015 and it was closed in March 2014 for Zhai et al. [26]. Further study is needed in the future focusing on the occurrence of lung cancer in women never-smokers.

In contrast, via a manual search we were able to identify four studies that met the selection criteria of the systematic review by Zhai et al. [26], but were not included [36-39]. Of them, two studies [38,39] were published after their search was closed in March 2014, whereas two studies [36,37] were published before, which shows the importance of a hand search. Yu et al. [39] overlaps with two other studies presented by the same authors based on the same data sources [31,35], and thus, studies for a future meta-analysis should be selected carefully.

Lung tissues are known to have the highest sensitivity for HPV DNA detection [24], and six case-control studies were excluded from this meta-analysis because HPV infection was tested using DNA or antibody samples taken from bronchoalveolar lavage fluid or blood [40-45]. Using blood in lieu of tissue to test for HPV infection has the potential to be used as an early screening tool for lung cancer in women never-smokers [2]. In addition, there is some evidence that taking oral contraceptives is related to HPV proliferation [46], and thus, future studies are needed on the occurrence of lung cancer in women taking oral contraceptives [47,48]. As lung cancer in never-smokers is anticipated to become a serious problem soon [2,49], efforts should be made from different approaches to establish preventive policies.

In order to prove that a specific virus causes cancer, a case-control study must be conducted in order to meet necessary standards [50,51]. However, tumor-based case-control studies have the drawback of frequent measurement errors [52,53]. To overcome the problem, adaptive systematic reviews need to be continuously conducted.

## Figures and Tables

**Table 1.** Summary of four selected case-control studies for association of HPV 16/18 infection with lung cancer in women and never-smokers

Author_year [Ref]	Nation	Specimen	Group	Case (n/N)	Control (n/N)	OR (95% CI)
Cheng_2001 [29]	Taiwan	PET	Women	27/45	2/11	6.75 (1.30, 34.94)
			Never-smokers	38/78	7/48	5.56 (2.23, 13.91)
Nadji_2007 [30]	Iran	PET	Women	6/25	2/23	3.32 (0.60, 18.45)
			Never-smokers	5/21	2/40	5.94 (1.04, 33.85)
Yu_2009 [31]	China	PET	Never-smoker	4/11	2/8	1.71 (0.23, 12.89)
Sarchianaki_2014 [32]	Greece	PET	Women	2/9	0/16	11.00 (0.47, 258.48)

Author_year [Ref], name of first author_year of publication [reference number].

HPV, human papillomavirus; PET, paraffin-embedded tissue; OR, odds ratio; CI, confidence interval.

**Table 2.** Human papillomavirus detection and risk of lung cancer in women and never-smokers

Type	Women SOR (95% CI) [I-squared, %]	Never-smokers SOR (95% CI) [I-squared, %]	Women & never-smokers AOR (95% CI)^1^
16/18	5.32 (1.75, 16.17)	4.78 (2.25, 10.15)	
	[0.0]	[0.0]	
16			3.98 (1.13, 13.98)
18			11.66 (2.94, 46.27)

SOR, summary odds ratio; CI, confidence interval; AOR, adjusted odds ratio.

1 Adjusted for age, tumor type, and tumor stage suggested by Cheng et al. [29].
