# Supplementary material for: Human papillomavirus infection and risk of lung cancer in never-smokers and women: an ‘adaptive’ meta-analysis
Source: Epidemiol Health. 2015 Nov 17;37:e2015052. doi: 10.4178/epih/e2015052 (PMC4722221; doi:10.4178/epih/e2015052)
Supplement: Supplementary file 1 [file epih-37-e2015052-supplementary.pdf]

<체계적 고찰>

인유두종 감염과 비흡연자 및 여성 폐암 발생 관련성

# : '개작' 메타분석

Human Papillomavirus Infection and Risk of Lung cancer in Never-smokers and Women : An 'Adaptive' Meta-Analysis

Abstract

Objectives: Occurrence of lung cancers in Koreans is increasing in women with never smoking history. Human papillomavirus (HPV) infection is suggested as a modifiable risk factor of lung cancer in never-smokers and women (LCNSW). This systematic review (SR) aimed to evaluate an association between HPV infection and lung cancer risk in LCNSW.

Methods: Based on a SR and some expert reviews, we made the list of refereed, cited, or related articles using the PubMed and Scopus databases. All of the case-control studies that obtained the odds ratio of HPV infection in LCNSW were selected. Estimate of summary odds ratio (SOR) with 95% confidence intervals (CI) were calculated.

Results: Total 4 case-control studies were finally selected. The fixed effect model was applied because of homogeneity (I-squared = 0.0%). The SORs in women and in non-smokers were 5.40 (95% CI: 1.80-16.23) and 4.81 (95% CI: 2.28-10.13), respectively.

Conclusions: These results showed a significant effect of HPV infection in LCNSW. It would be an evidence for necessary to set up a preventive plan against LCNSW.

Keyword: Lung neoplasms, Risk factor, Human Papillomavirus, Meta-analysis

Introduction

폐암 (lung cancer)은 한국인 암 사망의 수위를 차지하고 있어 질병부담이 가장 큰 원발암이다 [1]. 국가통계기관이 제공한 2002-2012년 폐암 발생현황을 살펴보았을 때, 여성에서의 발생률 증가와 함께 선암 (adenocarcinoma)의 비중이 높아졌다 [2]. 이런 사실은 한 지역 암센터에서 치료한 폐암환자들에서도 확인되며 [3], 특히 여성 폐암 대부분은 비흡연자들 (73.0%)로 보고하였다.

이렇게 여성 비흡연자의 폐암발생이 증가하는 추세는 전 세계적으로 나타나는 현상으로 [4-5], 비흡연자들의 폐암은 흡연자의 폐암과 다른 질병으로 보아야 한다는 주장이 제기되고 있다 [6-8]. 여성 비흡연자들의 폐암에 대한 원인 가설들 중 우선적으로 제기되는 것은 간접흡연 (second-hand smoking)이다 [6,7,9-11]. 그러나 아시아인의 비흡연 폐암 발생에서 간접흡연이 관여할 가능성이 낮다는 유전체연구 보고 [12]가 있을 뿐만 아니라, 간접흡연을 통제하는 것은 본인의 노력만으로는 어렵고 사회 공동체가 노력해야 한다는 점에서 암예방 대책에 한계가 있다. 대신 개입 가능 (modifiable)한 위험요인들 중의 하나로 인유두종바이러스 (human papilloma virus, HPV) 감염이다 [6,7,9]. 자궁경부암 [13], 전립선암[14], 유방암 [15] 등의 위험요인으로써, 현재 상용화된 예방 백신이 있기 때문이다 [16].

HPV DNA가 폐암 조직의 약 20%에서 검출되며 [17-20], 아시아인의 폐암 조직에서 더 높은 검출률을 보이고 있다 [21-24]. 한국인 폐암에서는 HPV 33아형의 검출률이 31.3%

임을 보고하였다 [25]. 2014년도에 발표된 체계적 고찰연구에 따르면 [26], HPV 감염으로 인한 발생 위험도 (odds ratio, OR)가 5.67배 (95% confidence intervals, CI: 3.09-10.40)이며, 아시아인들에서는 6.23배 (95% CI: 2.78-13.97)배로 다른 인종에 비하여 더 높았다. 그런데, 해당 연구에서 여성과 비흡연자에서의 위험도가 얼마인지에 대한 하부군 연구(sub-group analysis)는 제시하지 않았다. 따라서 본 연구의 목적은 여성 및 비흡연자에서 HPV 감염과 폐암발생의 관련성을 알아보는 체계적 고찰연구를 수행하는 것이다.

Subjects & Methods

가. 관련한 논문 검색 및 선정

기존에 수행된 체계적 고찰 논문을 검색에 최대한 활용한다는 점에서, 전자검색이 아닌 손 검색 (hand search) 전략을 적용하였다 [14, 27, 28]. 즉, 5개의 체계적 고찰 논문을 중심으로 참고문헌 (references)들을 탐색하고, 논문별로 PubMed (www.ncbi.nlm.nih.gov/pubmed)와 Scopus (www.elsevier.com/solutions/scopus) 데이터베이스가 제공하는 "Cited articles" 와 "Similar (Related) articles"의 목록을 확보하였다.

연구 목적을 위해 비흡연자 및 여성에 있어 HPV DNA 검사를 시행한 환자-대조군 연구가 선정 기준이다. 따라서, 우선 수합된 목록의 논문별로 초록 및 본문 내용에서 다음의 3가지 제외 조건들을 적용하였다. (1) 가설이 다른 경우 (2) 전문가 종설 혹은 체계적 고찰 (3) 환자사례연구. 이를 적용하고 남은 환자-대조군 연구 논문에 있어 (4) 환자-대조군연구이지만 병리조직에서 HPV DNA를 확인하지 않는 경우 (5) 환자-대조군연구이지만 여성 및 비 흡연자에 대한 정보를 얻어낼 수 없는 경우를 적용하여 분석 대상이 될 논문을 최종 선정하였다.

나. 통계분석

각 논문별로 제외 기준의 적용과 HPV 관련 정보 확보를 두 연구자가 진행하였다. 확보한 대상군과 HPV 양성자의 수치를 통해 OR과 95% CI을 구하였다. 이질성 여부 확인은 I-squared 값(%)을 기준으로 평가하였으며, 동질성이 확보될 경우 고정효과모형 (fixed effect model)에 따른 총교차비 (summary odds ratio, SOR)와 이의 95% CI을 구하는 메타분석을 시행하였다. 통계적 유의수준을 5%로 정했으며, MedCalc version 15.8 (www.medcalc.org) 통계프로그램을 이용하였다.

Results

Figure 1은 메타분석을 위한 최종 선정과정을 나타낸 것이다. HPV와 폐암간의 유병률 (prevalence) 과 위험도 (risk ratio) 를 알아보기 위하여 진행된 체계적 고찰 5편을 기준으로 이의 참고문헌 136편과, PubMed 와 Scopus를 활용한 인용 (cited)과 관련 (related) 문헌은 1,219편을 확보하였다. 총 1,355 편에 있어 선정 기준을 적용하였을 때, (1) 가설이 다른 경우 1,219편 (2) 전문가 종설 혹은 체계적 고찰 19편 (3) 환자사례연구 96편 (4) 환자-대조군연구이지만 병리조직에서 HPV DNA를 확인하지 않는 6편 (5) 환자-대조군연구이지만 여성 혹은 비 흡연에 대한 정보를 얻어낼 수 없는 11편을 제외시켰다. 따라서 총 1,351편이 제외되어 최종적으로 4편이 선정되었다 [29-32].

Table 1은 최종 선정된 4편의 환자-대조군 연구 논문에 있어, 대상자의 국적, 검사 시료, 여성 혹은 비흡연군에 따른 환자군과 대조군의 분포를 정리하였고, HPV DNA 양성 여부에 따라 산출된 OR 및 95% CI이다. 여성군과 비흡연군 각각 3 편의 정보를 가지고 있었으며, 두 군 모두에서 I-squared 값이 0%로 동질하였다 (Figure 2). Table 2는 HPV DNA 16/18 아형에 있어 고정효과모형을 적용한 메타분석 결과를 제시한 것이다. 여성군의 SOR은 5.40 (95% CI: 1.80-16.23)로, 비흡연군의 SOR 4.81 (95% CI: 2.28-10.13) 으로 통계적 유의성을 보였다.

Discussion

HPV 감염에 따른 폐암발생 SOR은 여성군 5.397, 비흡연군 4.806 로 산출되었다. 이는 남녀를 나누지 않을 경우 SOR 5.67과 유사한 수준이다 [26]. 추가로 여성 비흡연자에서 18아형 감염은 11.66배 (95% CI: 2.94-46.27) 위험하다는 보고 [29]를 감안하면 (Table 2), 실제의 위험도는 더 높을 것으로 예상된다. 이런 결과들에 따르면, HPV 감염을 예방하는 백신접종은 향후 여성의 폐암 발생까지도 줄일 수 있다는 근거가 된다. 또한, 남녀를 구분하지 않은 경우와 여자로 제한한 경우의 SOR이 유사한 사실에서, HPV 감염이 흡연 남성보다는 비흡연 여성의 폐암 발생에 더 큰 비중으로 작용한다고 유추할 수 있겠다 [33,34].

이번 체계적 고찰에 선정된 4편 [29-32]은 모두 환자-대조군 연구이었다. HPV DNA 실험을 위해서는 코호트 연구를 수행하기 어렵기 때문으로 판단된다. 선정된 4편 중 3편 [29,30,32]은 Zhai et al. [26] 논문의 분석대상으로 선정된 것이다. 나머지 Yu et al. [31] 논문은 같은 저자들의 2013년도 발표 논문 [35] 대신하여 선정된 것은, 자군에 대한 분석 정보를 갖고 있었기 때문이다. 이처럼 논문이 추가되지 않은 것은 본 연구의 검색 마감시점이 2015년 9월인 반면, Zhai et al. [26] 연구는 2014년 3월이기 때문이다. 향후 여성 비흡연자에서의 폐암 발생에 대한 연구가 보다 집중적으로 이루어질 필요가 있다.

한편 이번 손 검색을 통해 Zhai et al. [26] 체계적 고찰에서 선정기준에 맞지만 선정

되지 않은 논문 네 편을 확보할 수 있었다 [36-39]. 검색 종료인 2014년 3월을 기점으로 그 이후에 발표된 두 편 [38,39]이 있는 반면, 그 이전에 발표된 두 편 [36,37]이 있다는 점에서 손 검색의 중요성이 강조된다. 이중 Yu et al. [39] 은 동일한 자료원에서 동일한 저자가 발표했던 두 편의 논문 [31,35]과 중복되어, 향후 메타분석을 수행할 때 대상자 선정에 유의할 필요가 있다.

폐 조직에서의 HPV DNA 검출이 가장 민감한 것으로 알려진 가운데 [24], 기관지 세척액이나 혈액에서 DNA 혹은 항체 검출로 PV 감염 여부를 적용한 환자-대조군 연구 6편이 검색과정에서 분석 대상에서 제외되었다 [40-45]. 이처럼 조직이 아니라 혈액을 이용하여 HPV 감염여부를 알아보는 시도는, 향후 비흡연 여성에 있어 폐암의 조기 검진에도 활용할 가능성이 있겠다 [2]. 또한 경구 피임제 (oral contraceptives) 복용이 HPV 증식에 관여한다는 주장 [46]이 있어, 호르몬제 복용에 의한 폐암발생 위험에 대하여도 추가적인 연구가 필요하다 [47,48]. 향후 비흡연자의 폐암 발생이 심각한 문제가 될 것을 예상하고 [49], 이에 대한 예방대책을 수립하는 노력이 다각도로 있어야 할 것이다.

특정 바이러스가 암을 유발함을 입증하려면 필요한 기준들 [50]을 만족시키기 위해서는 환자사례 연구가 아니라 환자-대조군 연구를 수행해야 한다 [51]. 그러나 암조직을 활용한 환자-대조군 연구 (tumor-based case-control study)는 측정 오류를 범하기 쉽다는 단점이 있다 [52,53]. 이를 극복하기 위하여 지속적인 adaptive Systematic reviews (SR) 연구가

필요하다.

References

1. Jung KW, Won YJ, Kong HJ, Oh CM, Cho H, Lee DH, et al. Cancer statistics in Korea: incidence, mortality, survival, and prevalence in 2012. Cancer Res Treat 2015;47:127-141.
2. Bae JM. Modifiable risk factors of lung cancer in never-smoker women. Epidemiol Health 2015.
3. Kang HW, Park CW, Kim WJ, Song SY, Na KJ, Jeong JU, et al. Never-smoker lung cancer is increasing. J Lung Cancer 2012;11:89-93.
4. Alberg AJ, Samet JM. Epidemiology of lung cancer. Chest 2003;123:23S-49S.
5. Zhou W, Christiani DC. East meets West: ethnic differences in epidemiology and clinical behaviors of lung cancer between East Asians and Caucasians. Chin J Cancer 2011;30:287-292.
6. Subramanian J, Govindan R. Lung cancer in 'Never-smokers': a unique entity. Oncology 2010;24:29-35.
7. Alberg AJ, Wallace K, Silvestri GA, Brock MV. Invited commentary: the etiology of lung cancer in men compared with women. Am J Epidemiol 2013;177:613-616.
8. Sun S, Schiller JH, Gazdar AF. Lung cancer in never smokers—a different disease. Nat Rev Cancer 2007;7:778-790.
9. Planchard D, Besse B. Lung cancer in never-smokers. Eur Respir J 2015;45:1214-1217.
10. Molina JR, Yang P, Cassivi SD, Schild SE, Adjei AA. Non-small cell lung cancer: epidemiology, risk factors, treatment, and survivorship. Mayo Clin Proc 2008;83:584-594.
11. Lam WK. Lung cancer in Asian women-the environment and genes. Respirology 2005;10:408-417.
12. Krishnan VG, Ebert PJ, Ting JC, Lim E, Wong SS, Teo AS, et al. Whole-genome sequencing of asian lung cancers: second-hand smoke unlikely to be responsible for higher incidence of lung cancer among Asian never-smokers. Cancer Res 2014;74:6071-6081.

13. Wattleworth R. Human papillomavirus infection and the links to penile and cervical cancer. J Am Osteopath Assoc 2011;111:S3-S10.

14. Bae JM. Human papillomavirus 16 infection as a potential risk factor for prostate cancer: an adaptive meta-analysis. Epidemiol Health 2015;37:e2015005.

15. Bae JM. Two hypotheses of dense breasts and viral infection for explaining incidence of breast cancer by age group in Korean women. Epidemiol Health 2014;36:e2014020.

16. Lowy DR, Schiller JT. Reducing HPV-associated cancer globally. Cancer Prev Res 2012;5:18-23.

17. Giuliani L, Favalli C, Syrjanen K, Ciotti M. Human papillomavirus infections in lung cancer. Detection of E6 and E7 transcripts and review of the literature. Anticancer Res 2007;27:2697-2704.

18. Srinivasan M, Taioli E, Ragin CC. Human papillomavirus type 16 and 18 in primary lung cancers-a meta-analysis. Carcinogenesis 2009;30:1722-1728.

19. Hasegawa Y, Ando M, Kubo A, Isa S, Yamamoto S, Tsujino K, et al. Human papilloma virus in non-small cell lung cancer in never smokers: a systematic review of the literature. Lung Cancer 2014;83:8-13.

20. Klein F, Amin Kotb WF, Petersen I. Incidence of human papilloma virus in lung cancer. Lung Cancer 2009;65:13-18.

21. Syrjänen KJ. HPV infections and lung cancer. J Clin Pathol 2002;55:885-891.

22. Chen YC, Chen JH, Richard K, Chen PY, Christiani DC. Lung adenocarcinoma and human papillomavirus infection. Cancer 2004;101:1428-1436.

23. Subramanian J, Govindan R. Lung cancer in never smokers: a review. J Clin Oncol 2007;25:561-570.

24. Rezazadeh A, Laber DA, Ghim SJ, Jenson AB, Kloecker G. The role of human papilloma virus in

lung cancer: a review of the evidence. Am J Med Sci 2009;338:64-67.

25. Park MS, Chang YS, Shin JH, Kim DJ, Chung KY, Shin DH, et al. The prevalence of human papillomavirus infection in Korean non-small cell lung cancer patients. Yonsei Med J 2007;48:69-77.

26. Zhai K, Ding J, Shi HZ. HPV and lung cancer risk: a meta-analysis. J Clin Virol 2015;63:84-90.

27. Bae JM. Narrative reviews. Epidemiol Health 2014;36:e2014018.

28. Bae JM. The necessity of an observational study on the interactions between allergic history and citrus fruit intake for the prevention of pancreatic cancer. Epidemiol Health 2015;37:e2015028.

29. Cheng YW, Chiou HL, Sheu GT, Hsieh LL, Chen JT, Chen CY, et al. The association of human papillomavirus 16/18 infection with lung cancer among nonsmoking Taiwanese women. Cancer Res 2001;61:2799-2803.

30. Nadji SA, Mokhtari-Azad T, Mahmoodi M, Yahyapour Y, Naghshvar F, Torabizadeh J, et al. Relationship between lung cancer and human papillomavirus in north of Iran, Mazandaran province. Cancer Lett 2007;248:41-46.

31. Yu Y, Yang A, Hu S, Yan H. Correlation of HPV-16/18 infection of human papillomavirus with lung squamous cell carcinomas in Western China. Oncol Rep 2009;21:1627-1632.

32. Sarchianaki E, Derdas SP, Ntaoukakis M, Vakonaki E, Lagoudaki ED, Lasithiotaki I, et al. Detection and genotype analysis of human papillomavirus in non-small cell lung cancer patients. Tumour Biol 2014;35:3203-3209.

33. Gazdar AF, Thun MJ. Lung cancer, smoke exposure, and sex. J Clin Oncol 2007;25:469-471.

34. Thun MJ, Hannan LM, Adams-Campbell LL, Boffetta P, Buring JE, Feskanich D, et al. Lung cancer occurrence in never-smokers: an analysis of 13 cohorts and 22 cancer registry studies. PLoS Med 2008;5:e185.

35. Yu Y, Yang A, Hu S, Zhang J, Yan H. Significance of human papillomavirus 16/18 infection in association with p53 mutation in lung carcinomas. Clin Respir J 2013;7:27-33.

36. Krikelis D, Tzimagiorgis G, Georgiou E, Destouni C, Agorastos T, Haitoglou C, et al. Frequent presence of incomplete HPV16 E7 ORFs in lung carcinomas: memories of viral infection. J Clin Virol 2010;49:169-174.

37. Galvan A, Noci S, Taverna F, Lombardo C, Franceschi S, Pastorino U, et al. Testing of human papillomavirus in lung cancer and non-tumor lung tissue. BMC Cancer 2012;12:512.

38. Anantharaman D, Gheit T, Waterboer T, Halec G, Carreira C, Abedi-Ardekani B, et al. No causal association identified for human papillomavirus infections in lung cancer. Cancer Res 2014;74:3525-3534.

39. Yu Y, Liu X, Yang Y, Zhao X, Xue J, Zhang W, et al. Effect of FHIT loss and p53 mutation on HPV-infected lung carcinoma development. Oncol Lett 2015;10:392-398.

40. Papadakis ED, Soulitzis N, Spandidos DA. Association of p53 codon 72 polymorphism with advanced lung cancer: the Arg allele is preferentially retained in tumours arising in Arg/Pro germline heterozygotes. Br J Cancer 2002;87:1013-1018.

41. Carpagnano GE, Koutelou A, Natalicchio MI, Martinelli D, Ruggieri C, Di Taranto A, et al. HPV in exhaled breath condensate of lung cancer patients. Br J Cancer 2011;105:1183-1190.

42. Chiou HL, Wu MF, Liaw YC, Cheng YW, Wong RH, Chen CY, et al. The presence of human papillomavirus type 16/18 DNA in blood circulation may act as a risk marker of lung cancer in Taiwan. Cancer 2003;97:1558-1563.

43. Jain N, Singh V, Hedau S, Kumar S, Daga MK, Dewan R, et al. Infection of human papillomavirus type 18 and p53 codon 72 polymorphism in lung cancer patients from India. Chest. 2005;128:3999-4007.

44. Buyru N, Altinisik J, Isin M, Dalay N. p53 codon 72 polymorphism and HPV status in lung cancer.

Med Sci Monit 2008;14:CR493-CR497.

45. Simen-Kapeu A, Surcel HM, Koskela P, Pukkala E, Lehtinen M. Lack of association between human papillomavirus type 16 and 18 infections and female lung cancer. Cancer Epidemiol Biomarkers Prev 2010;19:1879-1881.

46. Efird JT, Toland AE, Lea CS, Phillips CJ. The combined influence of oral contraceptives and human papillomavirus virus on cutaneous squamous cell carcinoma. Clin Med Insights Oncol 2011;5:55-75.

47. Gasperino J, Rom WN. Gender and lung cancer. Clin Lung Cancer 2004;5:353-359.

48. Bae JM, Kim EH. Hormonal replacement therapy and risk of lung cancer in women: an adaptive meta-analysis of cohort studies. J Prev Med Public Health 2015. (in press)

49. Wakelee HA, Chang ET, Gomez SL, Keegan TH, Feskanich D, Clarke CA, et al. Lung cancer incidence in never smokers. J Clin Oncol 2007;25:472-478.

50. Joshi D, Buehring GC. Are viruses associated with human breast cancer? Scrutinizing the molecular evidence. Breast Cancer Res Treat 2012;135:1-15.

51. Liang W1, Tian H. Hypothetic association between human papillomavirus infection and breast carcinoma. Med Hypotheses 2008;70:305-7.

52. Engels EA, Wacholder S, Katki HA, Chaturvedi AK. Tumor-based case-control studies of infection and cancer: muddling the when and where of molecular epidemiology. Cancer Epidemiol Biomarkers Prev 2014;23:1959-64.

53. Zhai K, Ding J, Shi HZ. Author's reply to "comments on HPV and lung cancer risk: a meta-analysis" J Clin Virol 2015;63:92-3.
